# Usability Validation of an Integrated Hemodynamic and Pulmonary Monitoring System Using Eye-Tracking Analysis

**DOI:** 10.3390/jcm15072474

**Published:** 2026-03-24

**Authors:** Hyunju Jeong, Hyeonkyeong Choi, Hyungmin Kim, Wonseuk Jang

**Affiliations:** 1 Department of Medical Device Engineering and Management, Yonsei University College of Medicine, Seoul 06229, Republic of Korea; guswn99224@naver.com (H.J.); hyeonkyeong97@daum.net (H.C.);; 2 Medical Device Usability Research Center, Gangnam Severance Hospital, Yonsei University College of Medicine, Seoul 06230, Republic of Korea

**Keywords:** hemodynamics monitoring, lung ultrasound monitoring, usability test, eye tracker

## Abstract

**Background/Objectives**: Hemodynamic monitoring is essential for guiding appropriate treatment by assessing cardiac output and volume status, as well as for preventing complications associated with excessive fluid administration. The EdgeFlow CW10 Plus is a device that extends conventional hemodynamic monitoring by incorporating pulmonary abnormality surveillance through B-line detection. This study aimed to evaluate whether the hemodynamic monitoring and pulmonary monitoring functions are well integrated, and verify the usability and efficiency of the system. **Methods**: A usability test was conducted with a panel of 15 medical professionals from diverse specialties and varying levels of clinical experience. Data from satisfaction surveys, heat maps, the System Usability Scale (SUS), and the NASA-TLX were analyzed to determine whether usability differences existed based on the duration of clinical experience. **Results**: The device demonstrated a high overall task success rate, averaging 93.2%. Regarding eye-tracking analysis based on clinical experience, it was observed that participants with more years of experience either failed to direct their gaze toward task-relevant user interface (UI) elements as effectively as those with fewer years of experience or showed similar patterns. **Conclusions**: The usability evaluation confirmed that the hemodynamic and pulmonary monitoring functions of the EdgeFlow CW 10 PLUS are well integrated, with the device demonstrating high usability and satisfaction. This integration is expected to support medical professionals in monitoring cardiac output and fluid status, facilitating timely therapeutic interventions while preventing complications related to fluid overload.

## 1. Introduction

### 1.1. Integrated Hemodynamic and Pulmonary Monitoring System

Hemodynamic monitoring is a critical component in perioperative anesthesia and critical care settings as it enables clinicians to assess patient status and determine appropriate treatment [1,2,3]. Traditionally, invasive methods such as central venous and pulmonary artery catheterization, as well as minimally invasive approaches including arterial catheterization, have been widely used. However, these methods are associated with prolonged insertion times and an increased risk of procedure-related complications. Consequently, non-invasive hemodynamic monitoring has gained increasing preference in recent years [1]. Hemodynamic monitoring is particularly essential in the management of shock, a frequent occurrence in critically ill patients.

Shock is a life-threatening state of circulatory failure characterized by inadequate tissue perfusion and an imbalance between oxygen demand and supply [4,5]. Shock is broadly classified into hypovolemic, distributive, cardiogenic, and obstructive shock based on its underlying pathophysiology [4]. Fluid therapy serves as the primary treatment for these patients to improve tissue perfusion by increasing cardiac output and oxygen delivery [6]. While appropriate fluid therapy can be lifesaving, excessive administration may lead to severe complications, such as pulmonary edema, hypoxia, and organ dysfunction due to fluid overload [7,8]. Therefore, it is critical for medical professionals to utilize hemodynamic monitoring to assess cardiac output, fluid status, and clinical instability, enabling timely interventions and preventing complications associated with over-resuscitation [1,7].

One of the key indicators for monitoring fluid overload is the B-line. A B-line is defined as a vertical reverberation artifact originating from the pleural line caused by multiple reflections of air and liquid within the interlobular septa thickened by fluid [9,10,11]. The detection of B-lines is essential for evaluating pulmonary edema, not only in patients with cardiac and pulmonary diseases but also in those undergoing major surgery [10].

Traditionally, pulmonary edema was diagnosed via chest X-ray, which required repeated imaging at short intervals to monitor treatment efficacy [10]. Recently, however, lung ultrasound (LUS) has gained clinical favor for its ability to provide rapid visual assessment of lung tissue and the pleura without radiation exposure [10]. B-lines, which assist in diagnosing pulmonary edema, can be evaluated through LUS; they provide direct imaging of extravascular lung water (EVLW), a physiological variable with well-established diagnostic and prognostic value [11]. The potential of lung ultrasound can thus be extended to the hemodynamic management of critically ill patients [12].

This study evaluates the usability of the EdgeFlow CW10 Plus, an integrated hemodynamic and pulmonary monitoring system designed for use in perioperative and critical care environments. The device combines ultrasound-based hemodynamic monitoring with automated lung ultrasound analysis to support clinical assessment of circulatory status and pulmonary fluid accumulation. By integrating these monitoring functions into a single system, the device aims to assist clinicians in making timely clinical decisions during the management of critically ill patients.

**Figure 1:**
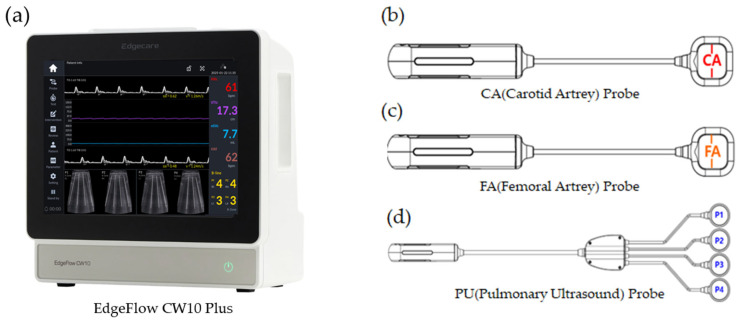
EdgeFlow CW10 Plus (EDGECARE INC., Yeongdeungpo-gu, Republic of Korea): (**a**) system; (**b**) CA probe; (**c**) FA probe; (**d**) PU probe.

The EdgeFlow CW 10 Plus, which integrates hemodynamic and pulmonary monitoring, consists of a main system (Figure 1a) and three specialized probes. As shown in Figure 1b,c, the CA probe (carotid artery) and FA probe (femoral artery) provide B-mode images to identify vessel locations, enabling blood flow velocity measurement via Doppler ultrasound once the probe head is secured to the target area. To ensure precise measurement of hemodynamic parameters, these probes feature an ‘Alignment Line’ on the upper surface of the probe head to assist medical professionals in accurate positioning. The PU probe, illustrated in Figure 1d, is composed of four heads and periodically acquires B-mode lung ultrasound (LUS) images from the attached pulmonary regions. The built-in software automatically detects B-lines and displays them on the lung ultrasound images, and the number of detected B-lines is displayed numerically.

**Figure A1:**
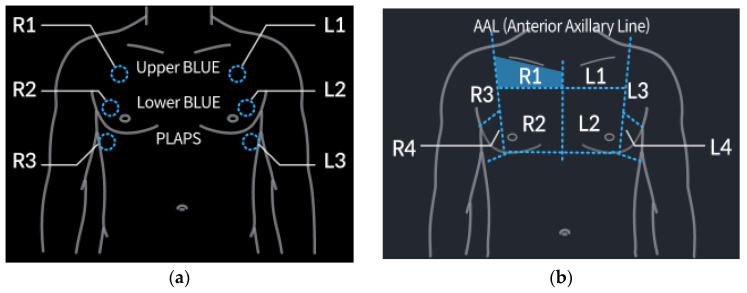
EdgeFlow CW10 Plus GUI: (**a**) 6-zone protocol; (**b**) 8-zone protocol.

When attaching the PU probe, the evaluator selects either the 6-zone protocol (Figure A1a) or the 8-zone protocol (Figure A1b), depending on the specific pulmonary regions to be assessed. Once hemodynamic and pulmonary monitoring commences, the trends and values of hemodynamic parameters become available for review. Simultaneously, medical professionals can monitor B-mode lung ultrasound (LUS) images and B-line detections captured through the four attached PU probe heads.

The EdgeFlow CW 10 Plus is a medical device that integrates pulmonary abnormality monitoring via B-line detection into conventional hemodynamic monitoring. The primary objective of this study was to evaluate the usability and operational effectiveness of the EdgeFlow CW10 Plus as an integrated hemodynamic and pulmonary monitoring system [13]. To this end, a usability test was conducted in a simulated environment using a manikin, involving medical professionals who are the actual clinical users of the device. Furthermore, eye-tracking glasses were utilized to analyze differences in visual search patterns according to clinical experience levels during device operation.

### 1.2. Eye Tracking

Eye tracking, also known as oculography, is frequently employed to evaluate the quality of user interfaces [14]. In the medical field, this technology has been widely utilized for usability evaluations of various medical device interfaces, such as ICU ventilators [15], and patient monitoring systems [16]. The eye-tracking method monitors the evaluator’s eye movements within a captured visual environment using an eye-tracker [17]. This approach allows for the analysis of visual perception and cognitive processes, complementing their subjective reports [14].

Eye movements are the result of complex cognitive processes that include target selection and movement planning and execution [18]. Through eye movement analysis, researchers can obtain objective and quantitative information regarding the quality, predictability, and consistency of these underlying cognitive processes [18,19]. The previous literature [20,21] has discussed various eye-tracking measurement techniques and research applications. The primary eye movement analysis techniques introduced in prior studies are summarized in Table 1 [18].

## 2. Materials and Methods

### 2.1. Participants

For the summative evaluation, we recruited medical professionals who are the intended users of the integrated hemodynamic and pulmonary monitoring system. The intended users of this device are healthcare professionals involved in hemodynamic monitoring and critical care management. In clinical practice, the device may be used in various clinical environments, including intensive care units (ICUs), emergency departments (EDs), and operating rooms. Therefore, to better reflect real-world clinical settings and identify potential use-related issues arising from different clinical workflows, participants were recruited from multiple specialties, including critical care medicine, anesthesiology, and emergency medicine.

The medical device usability standard IEC TR 62366-2 recommends a minimum of 15 participants when conducting usability testing as a summative evaluation [26]. This recommendation is further supported by the statistical rationale outlined in Annex K of IEC TR 62366-2, which demonstrates that when the probability of a usability deficiency occurring for a given participant is as low as 15%, a sample of 15 participants yields a probability of at least 91% of detecting at least one such deficiency. In accordance with this recommendation, the evaluation was conducted with 15 medical professionals.

This study was approved by the Institutional Review Board (IRB) of Gangnam Severance Hospital, Yonsei University Health System, and conducted in accordance with all guidelines and regulations relevant to the research methodology. Informed consent was obtained from all participants prior to their involvement, ensuring that ethical standards were maintained throughout the usability evaluation process (project no.: 3-2025-0229).

### 2.2. Study Procedures

#### 2.2.1. Testing Procedure

The summative evaluation was conducted as a usability test with medical professionals. Usability engineering encompasses activities throughout the entire product development lifecycle, with summative evaluation serving as the final pre-market verification step to confirm that intended users can safely and effectively operate the device.

In this study, the evaluation was conducted with intended users who had completed standardized training but were using the device for the first time, reflecting real-world clinical scenarios in which newly introduced medical devices are adopted. This approach differs from long-term familiarity evaluations, which assess proficiency developed through repeated use.

Consistent with IEC 62366-1, the evaluation focused on usability at the user interface level, specifically, whether intended users could safely and effectively operate the integrated hemodynamic and pulmonary monitoring functions within a single workflow without use errors, rather than assessing the physiological accuracy of monitoring outputs.

The usability test was conducted at a medical device usability research center, where all participants followed a standardized procedure. Prior to the arrival of each participant, the test administrator verified the device status and prepared the testing environment to closely simulate an actual clinical setting. To replicate an operating room, where hemodynamic monitoring devices are commonly used, the environment was equipped with a surgical bed and a patient dummy. The environmental conditions were maintained according to operating room standards, with the temperature set between 18 °C and 23 °C and humidity kept between 30% and 60%.

Upon arrival, the test administrator introduced the study and explained the objectives and procedures, after which informed consent was obtained. A pre-evaluation survey was then conducted to collect demographic and professional data, including age, specialty, clinical experience, and prior experience with similar products. Following the survey, the test administrator provided standardized device training. According to IEC 62366-1:2015 + AMD1:2020, Subclause 5.7.3 (Summative Evaluation Planning), when user training is provided prior to device use as part of realistic use conditions, an appropriate elapsed time should be allowed to accommodate potential learning decay before conducting the summative evaluation. Consistent with this recommendation, a 10 min washout period was introduced before the usability test to prevent participants from relying solely on short-term memory immediately after training. During this period, participants were encouraged to freely interact with the device.

During the usability test, participants performed the assigned tasks while wearing Tobii Pro Glasses 3 (Tobii Technology, Danderyd, Sweden), a wearable glass-type eye-tracking system, to record and analyze their visual search patterns.

**Figure A2:**
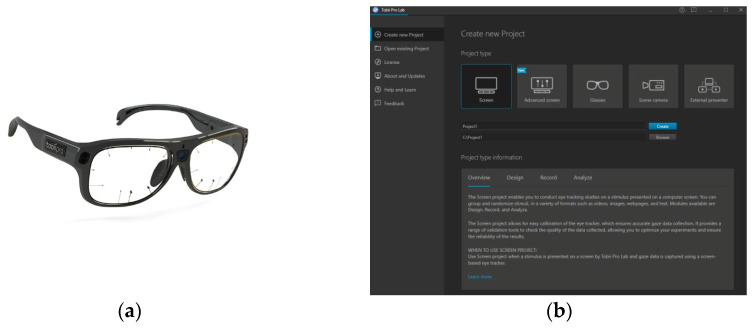
Eye tracker system: (**a**) Tobii Pro Glasses 3; (**b**) Tobii Pro Lab image.

The Tobii Pro Glasses 3 system consists of a head unit, a recording unit, and a controller application, with eye-tracking data analyzed using Tobii Pro Lab software(version 25.7; Tobii Technology, Danderyd, Sweden) [27] (see Appendix A, Figure A2 for device and software interface). Utilizing the Tobii Pro Glasses 3 allows for the collection of critical data from the evaluator’s perspective, which can be applied to enhance user experience and optimize the product [27]. Following the completion of the usability test, participants performed a satisfaction survey and an interview regarding the EdgeFlow CW 10 Plus.

#### 2.2.2. Use Scenarios for Summative Evaluation

The EdgeFlow CW 10 Plus supports the assessment of hemodynamic and fluid status through non-invasive ultrasound methods and provides a function to assist in detecting pulmonary abnormalities by identifying the number of B-lines from ultrasound images. Accordingly, two evaluation scenarios were developed to assess the device across various patient populations based on its functional capabilities.

Hypovolemic shock and pulmonary edema were selected as evaluation scenarios because they represent two physiologically contrasting hemodynamic conditions. Hypovolemia is characterized by reduced blood circulating volume and decreased preload, whereas pulmonary edema is associated with fluid overload and increased cardiac filling pressure. These two conditions therefore represent opposite ends of the fluid status spectrum commonly encountered in critical care settings.

In addition, the system evaluated in this study is not intended for the diagnosis of a specific disease but rather to support clinicians by providing hemodynamic and pulmonary monitoring information. Accordingly, these scenarios were designed to evaluate whether healthcare professionals could effectively review and utilize the information provided by the system under different hemodynamic conditions.

Scenario 1 established the baseline usability of hemodynamic monitoring using the CA and FA probes. Scenario 2 extended this workflow by removing the FA probe, retaining the CA probe, and adding the PU probe, reflecting a realistic clinical progression in which pulmonary monitoring is incorporated into an ongoing hemodynamic assessment.

Scenario 1 (Patient 1) simulated a patient with hypovolemia due to sepsis, requiring a fluid responsiveness test to determine whether to proceed with fluid administration. Scenario 2 (Patient 2) simulated a situation where pulmonary monitoring is necessary to prevent the occurrence of pulmonary edema following treatment for heart failure.

Within the overall scenarios, a critical task was defined as any task that, if performed incorrectly or not at all, could cause or potentially lead to significant harm to the patient or the user. Based on the “Hazard-Related Use Scenario” documentation, tasks with a severity rating of 3 (Serious) or higher, as well as those designated by the company, were selected as critical tasks. Detailed use scenarios are presented in the Table 2 and Table 3.

### 2.3. Usability Test Metrics

#### 2.3.1. Satisfaction

Nielsen regards satisfaction as an essential attribute of usability and describes it as the pleasantness experienced while using a system [28,29]. Satisfaction can be measured by collecting users’ subjective opinions, and averaging responses from multiple participants allows these data to serve as an objective indicator of system use [28]. The reason satisfaction is considered an attribute of usability is that it allows for an evaluation of whether users overall prefer the system [28]. For this reason, direct satisfaction surveys are widely used in usability studies [28].

Accordingly, in this usability test, a satisfaction survey was conducted following the completion of the tasks to assess participants’ satisfaction with the medical device for each scenario. The satisfaction survey utilized a 5-point Likert scale. In this scale, 5 points represented “Very Satisfied” and 1 point represented “Very Dissatisfied.” A score closer to 5 indicates that the participant was more satisfied.

#### 2.3.2. Heat Map

Among various eye-tracking analysis methods, this study utilized heatmaps. Heat map analysis is a technique used to analyze the spatial distribution of gaze, and it can be employed to analyze the gaze movements of individual participants as well as the aggregate gaze patterns of multiple participants [18]. A heat map is a graphical representation of data density generated by applying the Kernel Density Estimation (KDE) method [30]. While heat map analysis does not record the sequence of gaze movements, it analyzes the spatial distribution of fixations [18,31].

In a heatmap, colors represent the frequency or duration of gaze fixation on specific areas [18]. Red indicates areas with a high fixation frequency, while the frequency decreases as the color transitions from yellow to green [32,33]. These distinct color variations clearly illustrate the distribution and characteristics of the data at a glance [33]. Heat maps are particularly useful as they enable the visual analysis of an evaluator’s interaction with a system, making them highly effective for studies comparing different groups and conditions [18].

#### 2.3.3. System Usability Scale (SUS)

The System Usability Scale (SUS) is a subjective usability metric that can be quickly administered after a user completes assigned tasks [34]. As shown in Table 4, the SUS survey consists of 10 items responded to using a 5-point Likert scale [35]. These 10 items allow evaluators to rapidly assess the usability of a product without the need for complex analysis [35].

For the odd-numbered items (1, 3, 5, 7, and 9), which are positive statements, the score is calculated by subtracting the scale’s minimum value 1 from the user’s response. Conversely, for the even-numbered items (2, 4, 6, 8, and 10), which are negative statements, the score contribution is calculated by subtracting the user’s response from the scale’s maximum value of 5. The final SUS score, ranging from 0 to 100, is determined by summing the score contributions of all ten items and multiplying the total by 2.5 [35,36].

A SUS score of 68 represents the 50th percentile, indicating an average level of usability, while a score of 70 signifies “just about right” usability. Furthermore, a SUS score of 82 (±5) suggests that users are likely to be “promoters” who would recommend the system or product to others [35].

#### 2.3.4. NASA-TLX

The NASA-TLX (Task Load Index) is a subjective workload assessment scale originally developed for the aviation industry and is now actively utilized in the medical field [37]. In this evaluation, NASA-TLX metrics were employed to quantify the level of workload experienced by the intended users while operating the device. Following the completion of the evaluation tasks, participants respond to six items via an online questionnaire using a 20-point scale (low = 1, high = 20).

As shown in Table 5, the NASA-TLX consists of six dimensions that measure the user’s workload regarding mental demand, physical demand, temporal demand, performance, effort, and frustration [37,38]. The NASA-TLX scores are converted to a scale of 0 to 100 by subtracting 1 from the response score and multiplying it by 5 [36]. The descriptions for the six items are as follows [39].

### 2.4. Statistical Analysis

For the usability evaluation conducted with medical professionals, task success rates were categorized into three groups: Completed (C), Completed with Issues (CI), and Not Completed (NC). The task success rate was calculated as a percentage using Equation (1). This represents the ratio of tasks successfully finished—either independently (C) or with minor issues (CI)—relative to the total number of attempts.

(1) $$Task\;success\;rate\left(\%\right)=\frac{C+CI}{P_{n}}\times 100$$

To investigate device usability and satisfaction according to clinical experience, the 15 evaluators were divided into two groups based on their years of clinical practice: those with less than 10 years of experience (Group 1, *n* = 7) and those with 10 years or more (Group 2, *n* = 8). The cutoff of 10 years was selected to reflect the distribution of clinical experience among the participants (mean clinical experience: 10.13 years) and enable comparison between relatively less experienced and more experienced clinicians.

For the gaze data analysis, heat maps were generated using Tobii Pro Lab (version 25.7; Tobii Technology, Danderyd, Sweden). To analyze the differences in task performance based on experience, heat map analysis focused primarily on tasks with low success rates.

Statistical analyses for satisfaction, SUS, and NASA-TLX scores according to clinical experience were performed using SPSS software (version 31.0; IBM Corp., Armonk, NY, USA). Normality testing was performed prior to statistical analysis. Variables that satisfied the normality assumption were analyzed using an independent samples *t*-test, whereas variables that did not satisfy the normality assumption were analyzed using the Mann–Whitney U test. The statistical significance level was set at *p* < 0.05.

## 3. Results

### 3.1. Participant Statistics

A total of 15 medical professionals who met the eligibility criteria described in Section 2.1 (Participants) participated in this study. Participants were recruited through recruitment emails sent to medical staff at the primary and affiliated institutions, as well as to members of the Korean Society of Critical Care Medicine and the Korean Society of Emergency Medicine. The participants consisted of nine physicians and six nurses from anesthesiology (*n* = 5), emergency medicine (*n* = 2), and critical care medicine (*n* = 8). Participants had an average of 10.13 years of clinical experience, and eight participants had more than 10 years of clinical experience. The sociodemographic characteristics of the participants are summarized in Table 6.

### 3.2. Task Success Rate

In this study, 15 participants performed a total of 65 tasks across 14 use scenarios, as presented in Table 2 and Table 3. The overall average task success rate for all 65 tasks was 93.2%. In addition, the average task success rate for each individual use scenario exceeded the predefined task success criterion of 80%. The task success and failure rates for each use scenario are summarized in Table 7.

The use scenarios with the lowest success rates were alarm setting and review, with each recorded at 84.8%. Additionally, the newly added PU monitoring functions showed high success rates, with 97.0% for the PU ultrasound attachment scenario and 93.3% for the PU monitoring scenario.

As a result of analyzing the individual success rates for all tasks, five tasks were identified that did not meet the predefined task success criterion of 80%. Among these, one task was classified as a critical task, while the remaining four were non-critical tasks. The success and failure rates for these five tasks(Table 8) were further analyzed based on the participants’ years of clinical experience.

### 3.3. Eye Tracker Analysis

Visual search strategy differences according to clinical proficiency were analyzed for the five tasks that did not meet the test objective. To this end, we compared and analyzed the heat maps, focusing on the failure cases of Group 1 (less than 10 years of clinical experience) and Group 2 (10 years or more of clinical experience). Previous studies have suggested that clinical expertise typically develops after prolonged clinical practice, often requiring approximately 10 years of experience to establish stable diagnostic reasoning and decision-making patterns [40]. In addition, physicians’ clinical experience plays a critical role in clinical judgment and decision-making processes, with accumulated experience contributing to the development of expert-level cognitive strategies [40,41].

**Figure 2:**
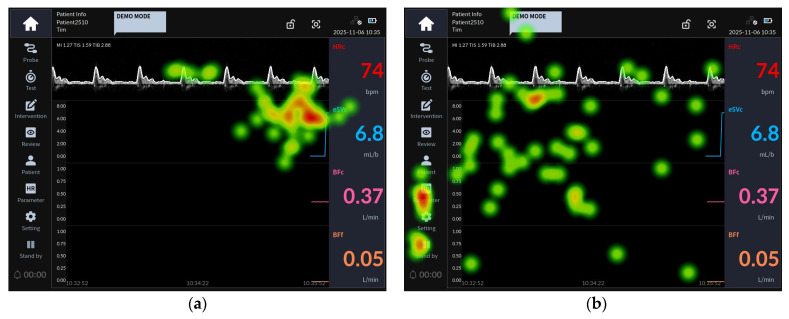
Task 26 representative heat map images: (**a**) eye-tracking heat map of the Group 1 representative user for task 26 failure; (**b**) eye-tracking heat map of the Group 2 representative user for task 26 failure.

Figure 2 presents the eye-tracking images of participants from Groups 1 and 2 who failed Task 26, which involved identifying parameter values via the eSVs graph.

This task can be successfully completed by clicking the eSVc graph in the home screen’s graphic trend area to identify parameter values at a specific time point.

The heat map analysis revealed that, as shown in Figure 2a, although the fixations of Group 1 participants (less than 10 years of clinical experience) who failed the task were concentrated on the eSVc graph trend area, they did not recognize the need to click the graph. In contrast, for the failed participants in Group 2 (10 years or more of clinical experience), the gaze patterns in Figure 2b were not concentrated on the eSVc graph area but were instead dispersed across the entire screen.

**Figure 3:**
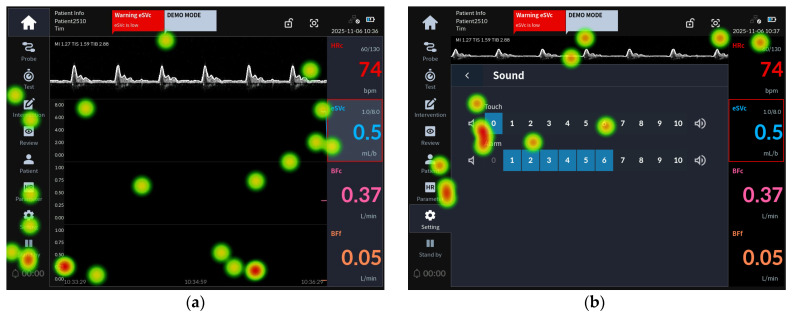
Task 34 representative heat map images: (**a**) eye-tracking heat map of the Group 1 representative user for task 34 failure; (**b**) eye-tracking heat map of the Group 2 representative user for task 34 failure.

Figure 3 presents the eye-tracking images of participants from Groups 1 and 2 who failed task 34, which involved pausing the alarm.

Task 34 was part of the alarm setting scenario and followed task 33 (change the alarm volume). The alarm pause button is accessible from both the home screen and the sound volume setting screen. Task 34 evaluated whether the user could activate the alarm pause button at the bottom of the main menu to suspend auditory alarms for two minutes.

In Group 1 failures, evaluators searched for the alarm pause button after returning from the alarm setting screen to the main screen, as shown in Figure 3a. In contrast, failed participants in Group 2 tended to search within the alarm setting screen where they had previously adjusted the volume, as illustrated in Figure 3b. Heat map analysis showed that Group 1 evaluators’ gaze was distributed around the alarm pause button, but they failed to perceive it. In Group 2, gaze remained concentrated on the volume adjustment area, with limited shifts toward the alarm pause button.

**Figure 4:**
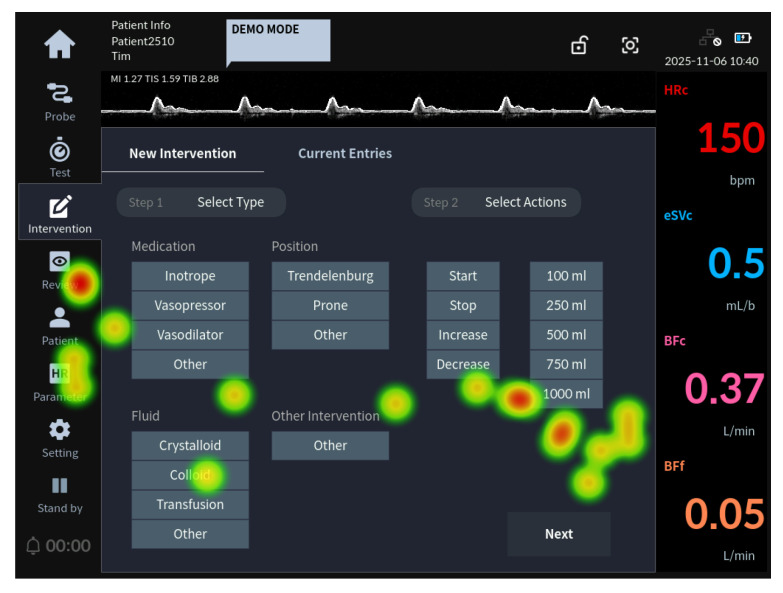
Task 41 representative heat map image: eye-tracking heat map of the Group 2 representative user for task 41 failure.

Regarding task 41, all Group 1 participants completed the task successfully; therefore, heat map analysis was not performed for this group. Figure 4 presents the eye-tracking image of a Group 2 participant who failed task 41 (recording the administration of 250 mL of colloid for hypovolemia treatment).

To complete task 41, the user needed to select ‘Colloid’ under the ‘Fluid’ category in Step 1 (select type) of the Intervention tab and then select “250 mL” in Step 2 (select actions).

As shown in Figure 4, the heat map analysis of evaluators in Group 2 who failed the task revealed that they identified “Colloid” in Step 1 but did not gaze at Step 2 or the “250 mL” option.

**Figure 5:**
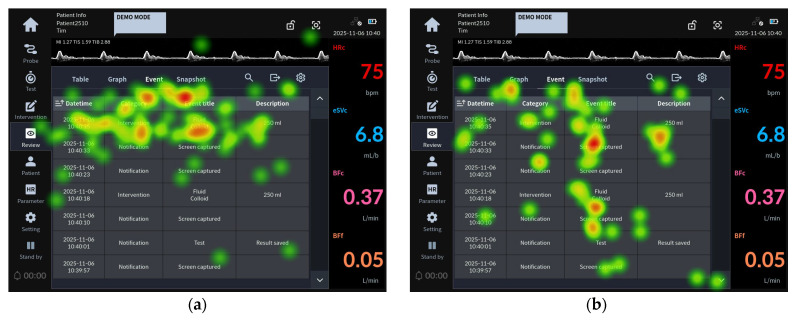
Task 43 representative heat map images: (**a**) eye-tracking heat map of the Group 1 representative user for task 43 failure; (**b**) eye-tracking heat map of the group 2 representative user for task 43 failure.

Figure 5 shows eye-tracking images of Group 1 and 2 participants who failed task 43, which involved verifying the fluid responsiveness test records on the Review screen.

To successfully complete task 43, users must verify the fluid responsiveness test record by identifying “Notification” in the Category and “Test” in the Event Title on the Event screen of the Review tab.

The heat map analysis revealed that participants in Group 1 directed attention to the Event Title, Category, and “Fluid Colloid” in the Event screen, as indicated by the colors in Figure 5a. In Figure 5b, the gaze of participants in Group 2 who failed the task was concentrated on the Screen Capture and “Fluid Colloid.” Successful completion of task 43 requires focusing on the word “Test” within the Event Title; however, participants in both groups primarily focused on “Fluid Colloid.”

**Figure 6:**
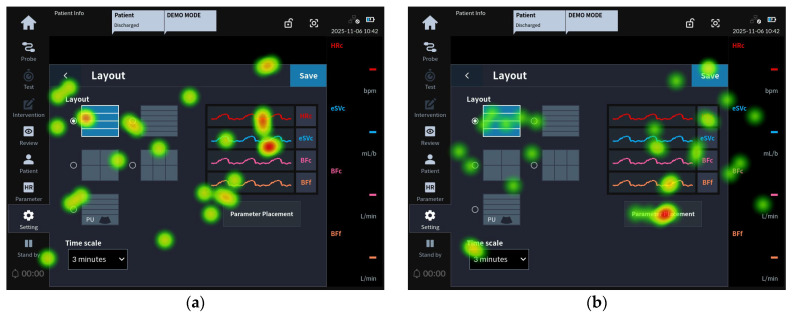
Task 51 representative heat map images: (**a**) eye-tracking heat map of the Group 1 representative user for task 51 failure; (**b**) eye-tracking heat map of the Group 2 representative user for task 51 failure.

Figure 6 presents the eye-tracking images of participants from Groups 1 and 2 who failed task 51, which required rearranging the home screen parameters in the following order: HRc, eSVc, VTIc, BFc, and B-line.

To successfully complete task 51, users needed to change the layout to one that included PU parameters in the Layout screen of the Settings tab. Subsequently, the user needed to change the order of the parameter layout by pressing the Parameter Placement button.

As shown in Figure 6a, the gaze of the participants in Group 1 who failed the task was focused on the existing 4-split layout and the image above the Parameter Placement button rather than the PU layout. According to Figure 6b, the gaze of the participants in Group 2 who failed the task was concentrated on the Parameter Placement button without locating the additional PU layout. This indicated a tendency where the gaze shift toward the additional PU layout was restricted.

### 3.4. Statistical Analysis Results

The 15 medical professionals who participated in this usability test were divided into two groups based on their clinical experience: Group 1 (less than 10 years, *n* = 7) and Group 2 (10 years or more, *n* = 8). We statistically analyzed the satisfaction, SUS, and NASA-TLX data for both groups. Prior to statistical analysis, normality testing was conducted for each variable. Variables that satisfied the normality assumption were analyzed using an independent samples *t*-test, whereas variables that did not satisfy the assumption were analyzed using the Mann–Whitney U test.

The statistical results according to clinical experience are presented in Table 9.

Statistical analysis of user satisfaction revealed high levels of satisfaction with the device regardless of clinical experience, with Group 1 (mean = 4.60, SD = 0.43) and Group 2 (mean = 4.51, SD = 0.49) showing no significant difference (*p* > 0.05). Similarly, the analysis of System Usability Scale (SUS) scores showed that usability was high for both groups, with Group 1 (mean = 86.79, SD = 15.39) and Group 2 (mean = 75.94, SD = 13.22) exhibiting no statistically significant difference (*p* > 0.05).

To compare the degree of workload during device use between the groups, each dimension of the NASA-TLX—mental demand, physical demand, temporal demand, performance, effort, and frustration—was statistically analyzed according to clinical experience. It was confirmed that there were no statistically significant differences in mental demand, temporal demand, performance, or frustration, regardless of clinical experience (*p* > 0.05).

However, significant differences were found in physical demand (*p* = 0.006) and effort (*p* = 0.040) according to clinical experience (*p* < 0.05). Physical demand was found to be higher in Group 2 (mean = 16.88, SD = 7.99) compared to Group 1 (mean = 2.86, SD = 7.56). Additionally, effort was higher in Group 2 (mean = 41.88, SD = 23.75) than in Group 1 (mean = 16.43, SD = 20.76).

## 4. Discussion

This study conducted a usability test with medical professionals on a hemodynamic monitoring device equipped with a newly added lung abnormality detection assistant function. Regarding the success rates of the new PU monitoring functions, high success rates were confirmed for prepare pulmonary monitoring (86.7%), PU probe attachment (97.0%), and PU monitoring (93.3%).

Based on the score for SUS Item 5 (I found the various functions in this system were well integrated, mean = 4.53), it was confirmed that the hemodynamic and lung ultrasound functions of the EdgeFlow CW10 Plus were well integrated. Furthermore, the score for SUS Item 6 (I thought there was too much inconsistency in this system, mean = 1.4) indicates that the newly added B-line detection-based lung abnormality monitoring function is highly consistent with the existing hemodynamic monitoring functions. During post-evaluation interviews, participants expressed satisfaction with the PU monitoring process and noted high usability, citing its similarity to other displays currently in use.

To analyze differences in visual search patterns according to clinical experience, heat maps were examined for failure cases associated with five tasks that did not meet the predefined task success criterion of 80%. For tasks 26 (check the parameter value through the graph of eSVc) and 34 (pause the alarm), Group 1 failed to complete the tasks even though their gaze reached the user interface elements. This suggests that a lack of visual emphasis on UI elements prevented cognitive recognition. Therefore, we propose improving the visibility of graph parameter values and the alarm pause button. In contrast, participants in Group 2 were either unable to direct their gaze toward relevant UI elements or showed dispersed gaze patterns across the screen. This suggests that clinicians with more than 10 years of clinical experience may be familiar with the user interfaces, layout structures, and operational workflows of monitoring devices commonly used in clinical practice, which hindered their ability to locate appropriate elements in a new interface environment.

For tasks 43 and 51, both groups exhibited similar causes for task failure. In task 43 (check the fluid responsiveness test record on the review screen), both groups focused on “Fluid Colloid” rather than the word “Test” within the “Event Title,” resulting in them checking intervention records instead of test records. Survey results indicated that categorizing events on the review screen would further improve visibility. Task 51 (change the parameter’s layout (placement) in order of HRc, eSVc, VTIc, BFc, and B-line) is a task that specifically involves adding the PU layout to monitor lung ultrasound B-mode images and B-line counts. The heat map analysis indicated that participants in both Group 1 and Group 2 did not immediately recognize the additional PU layout. This finding suggests that the layout modification required additional visual attention from users rather than indicating a fundamental usability issue with the integration of the monitoring functions. Since both groups struggled to identify the PU interface elements during the layout change process, UI improvements, such as providing greater visual emphasis on the PU interface, should be considered.

Regarding satisfaction with clinical experience, both groups showed high satisfaction with no significant difference (*p* > 0.05; Group 1: mean = 4.60, SD = 0.43; Group 2: mean = 4.51, SD = 0.49). Analysis of the SUS data revealed that Group 1 (mean = 86.79) and Group 2 (mean = 75.94) both scored above the average of 68. These scores exceeded the “Good” threshold of 70, indicating a satisfactory level of usability for both groups.

The NASA-TLX analysis showed that both groups experienced similar levels of workload with no significant difference (*p* > 0.05), as seen in the mean scores of Group 1 (mean = 14.64, SD = 10.02) and Group 2 (mean = 26.15, SD = 11.50). However, a statistically significant difference was found in the physical demand and effort subscales (*p* < 0.05). Specifically, Group 2 exhibited higher levels of physical demand (mean = 16.88, SD = 7.99) and effort (mean = 41.88, SD = 23.75).

This suggests that participants in Group 2, who had more than 10 years of experience, may have experienced higher physical demand and effort due to their familiarity with existing equipment interfaces. Nevertheless, high scores in Group 2 for SUS Item 3 (I thought the system was easy to use, mean = 4.12) and Item 9 (I felt very confident using the system, mean = 4.25) confirm that they still perceived the device as highly usable. This indicates that while workload was slightly higher due to habituation to previous systems, the overall perception of usability and satisfaction remained positive. It is expected that this workload will decrease as users become more accustomed to the evaluation device.

The present study confirmed that the hemodynamic and pulmonary monitoring functions of the EdgeFlow CW10 Plus are successfully integrated at the user interface level. The high task success rates and user satisfaction scores demonstrate that the integrated interface is operable and acceptable to intended users in a simulated clinical environment. These usability findings provide a foundation upon which future studies may examine the clinical effectiveness of this integrated monitoring system.

This study is significant for deriving real-world user experiences in a simulated clinical environment with 15 medical professionals from various specialties and experience levels. However, several limitations exist. First, although we gathered perspectives from various medical fields, the number of participants recruited was not uniform across all specialties. Second, due to technical limitations of the wearable eye-tracking device, eye-tracking data from one participant who wore glasses could not be reliably captured due to lens reflections. However, this participant successfully completed all tasks; therefore, their gaze data were not required for the analysis of visual search patterns associated with task failures.

In future research, we plan to recruit a more balanced number of participants across specialties and improve the eye-tracking setup by replacing the lenses of the eye-tracking glasses with prescription lenses. This approach would allow participants to use the device without wearing their own glasses and may improve the quality of gaze data collection.

## 5. Conclusions

The EdgeFlow CW 10 Plus is an integrated system that combines hemodynamic monitoring with lung monitoring through B-line detection. This study conducted a usability test with medical professionals to verify the functional integration and overall usability of the device. The analysis of overall task success rates showed that all use scenarios met or exceeded the predefined task success criterion of 80%, demonstrating a high level of usability of the device. For the five individual tasks that did not meet the predefined criterion, differences in visual search patterns according to clinical experience were further analyzed, with a focus on failure cases.

The heat map analysis indicated that visual search patterns between Group 1 (less than 10 years of clinical experience, *n* = 7) and Group 2 (10 years or more of clinical experience, *n* = 8) were generally similar. However, in some analyses, participants in Group 2 were less effective than those in Group 1 at directing their gaze toward task-related user interface elements.

Statistical analysis showed no significant differences between the groups regarding satisfaction (*p* = 0.536), SUS scores (*p* = 0.147), and NASA-TLX mean scores (*p* = 0.105), with all values exceeding *p* > 0.05. Nevertheless, Group 2 exhibited significantly higher levels of physical demand (*p* = 0.006) and effort (*p* = 0.040) on the NASA-TLX compared to Group 1.

Based on the heat map and statistical analyses, Group 2 demonstrated more dispersed gaze patterns and higher workload levels. These findings may reflect differences in visual search behavior between users with different levels of clinical experience when interacting with a new interface. Several factors may have contributed to the observed workload differences, including prior experience with conventional monitoring devices, visual acuity, physical fatigue, and adaptability to a new operational interface.

The usability evaluation confirmed that the EdgeFlow CW10 Plus demonstrated high usability and user satisfaction among intended users. The integration of hemodynamic and pulmonary monitoring functions was verified at the interface level, with all use scenarios meeting or exceeding the predefined task success criterion.

## Figures and Tables

**Table 1:** Key eye movement analysis metrics.

Category	Description
Area of Interest (AOI) Analysis	Area of Interest (AOI) analysis is a technique that assigns and analyzes eye movements within specific regions (or zones) of a visual scene [21,22]. Unlike measuring eye movements across the entire scene, AOI analysis provides eye-movement metrics confined to semantically specific areas, which is particularly useful for attention-based research [22].
Heat Map Analysis	Heat map analysis is a technique used to analyze the spatial distribution of eye movements across an entire visual scene. This technique can be applied to analyze the eye movements of individual participants as well as to comprehensively synthesize the collective eye movements of multiple participants.
Scanpath Analysis	A scanpath is a series of fixations and saccades that describe eye movement patterns during task performance [23]. Generally, a scanpath is visualized as a sequence of connected nodes (centers of fixation) and edges (saccades between two consecutive fixations) superimposed on the visual image of the scene [24,25].

**Table 2:** Scenario 1: fluid responsiveness test.

Use Scenario	Task No.	Task
[Scenario 1] A 27-year-old male patient (Patient 1) was observed to have low blood flow due to sepsis. Intravenous fluid administration is planned. Before administering the fluids, fluid responsiveness will be assessed to determine whether fluid therapy is indicated.		
Check the safety information	Task 1 *	Check ‘Caution’ in the Safety Information section in the user manual.
	Task 2	Check ‘PU probe attachment’ in the user manual.
	Task 3	Check the product’s ‘Alarm setting’ page in the user manual.
	Task 4	Check the product’s ‘Notification list’ page in the user manual.
Prepare for use	Task 5 *	Connect power cable to main system and check if the power cable has been properly connected. Place the cable in appropriate position.
	Task 6 *	Turn on the power on/off switch and press the power button.
	Task 7 *	Connect CA probe to main system.
	Task 8 *	Connect FA probe to main system.
Register Patient 1	Task 9	Admit a new patient. - Patient ID: Patient2510 - Patient Name: Tim - Sex: Male - Date of Birth: 26 June 1998 - Height (cm): 176 - Weight (kg): 75
CA probe, FA probe attachment	Task 10 *	Open the Probe menu.
	Task 11 *	Use palpation to find the location of the carotid artery in the patient’s neck and then clean the carotid artery area with an alcohol swab.
	Task 12 *	Remove the release film from one side of the gel pad and attach it evenly without air bubbles to the CA probe head.
	Task 13 *	Remove the release film on the other side of the gel pad attached to the probe head, apply sufficient pressure to the probe head, and attach it to the palpable carotid artery.
	Task 14 *	Press Scan button and check B-mode image.
	Task 15 *	Start CA monitoring.
	Task 16 *	Open the FA tab of the Probe menu.
	Task 17 *	Find the location of the femoral artery area and then clean the femoral artery area with an alcohol swab.
	Task 18 *	Remove the release film from one side of the gel pad and attach it evenly without air bubbles to the FA probe head.
CA probe, FA probe attachment	Task 19 *	Remove the release film on the other side of the gel pad attached to the probe head, apply sufficient pressure to the probe head, and attach it to the palpable femoral artery.
	Task 20 *	Press Scan button and check B-mode image.
	Task 21 *	Start FA monitoring.
Monitoring	Task 22	Adjust display brightness.
	Task 23	Change the parameter’s layout (placement) in order of HRc, eSVc, BFc, BFf.
	Task 24	Lock the current screen.
	Task 25	Unlock the screen.
	Task 26	Check the parameter value through the graph of eSVc.
	Task 27	Capture a snapshot.
Alarm setting	Task 28 *	Turn on the alarm of HRc and eSVc.
	Task 29 *	Change the HRc alarm range to 60–130.
	Task 30 *	Change the priority of HRc upper limit (Max.) to medium (yellow) and apply the changes.
	Task 31 *	Point the HRc alarm range (upper/lower limit) displayed on the Home screen.
	Task 32 *	Point the HRc or eSVc alarm message displayed on the Home screen.
	Task 33 *	Change the alarm volume.
	Task 34 *	Pause the alarm.
Fluid responsiveness test	Task 35 *	Conduct the fluid bolus test as follows: - Challenge duration: 1 min - Type of fluid: crystalloid - Volume of fluid: 100 mL
		Conduct the PLR Test as follows: Challenge duration: 1 min
	Task 36 *	Measure the baseline.
		Using the bed, raise the upper position of the body to 45 degrees and measure the baseline.
	Task 37 *	You have noticed the problem with the baseline measurement. Restart the baseline measurement.
		You have noticed the problem with the baseline measurement. Restart the baseline measurement.
	Task 38 *	If the measurement is sufficient, proceed to the next step.
	Task 39 *	Start fluid challenge.
		Lower the upper body and elevate the lower body to a 45-degree angle using the bed to begin the PLR test.
	Task 40 *	If the measurement is sufficient, check the result.
Review	Task 41	To improve hypovolemia, administer 250 mL of colloid and record this intervention.
	Task 42	Additionally, 250 mL of colloid was administered. Copy the previous intervention record and enter ‘f/u’ in the memo field.
	Task 43	Check the fluid responsiveness test record on the review screen.
	Task 44	Check the alarm records on the review screen.
	Task 45	Please check the Table Review to verify the previous values before exporting the current monitoring records.
	Task 46	Check the captured screen and enlarge it.
	Task 47	Insert the USB into the main system and export data.
Discharge Patient 1	Task 48	Remove the FA probe from the patient.
	Task 49	Discharge the patient.

* Tasks marked with an asterisk are those selected as critical tasks.

**Table 3:** Scenario 2: pulmonary monitoring for edema prevention.

Use Scenario	Task No.	Task
[Scenario 2] To prevent pulmonary edema following heart failure treatment in 28-year-old male patient (Patient 2), pulmonary monitoring is to be performed. (The attachment process of patient 2’s CA probe is omitted.)		
Prepare pulmonary monitoring	Task 50 *	Connect PU probe to main system.
	Task 51	Change the parameter’s layout (placement) in order of HRc, eSVc, VTIc, BFc, B-line.
Register Patient 2	Task 52	Admit a new patient. - Patient ID: Patient2511 - Patient Name: John - Sex: Male - Date of Birth: 26 July 1997 - Height (cm): 169 - Weight (kg): 65
PU probe attachment	Task 53 *	Open the PU tab of the Probe menu.
	Task 54	Clean the designated chest area where the probe will be attached with an alcohol swab.
	Task 55 *	Please select the protocol to be applied. - 6-Zone - 8-Zone
	Task 56 *	Remove the release film from one side of the gel pad and attach it evenly without air bubbles to each PU probe head.
	Task 57 *	Remove the release film on the other side of the gel pad attached to each probe head and attach the probe head to the patient’s body according to the area specified in the selected protocol.
	Task 58 *	Match each probe head with the corresponding probe position where the probe head is attached.
	Task 59 *	Press Scan button and check pulmonary B-mode image.
	Task 60 *	Starting from P1, check whether the B-mode image appears for all PU probe heads.
	Task 61 *	Start B-line monitoring.
PU monitoring	Task 62 *	Change the color of the B-line parameter value to yellow.
Discharge Patient 2	Task 63	Remove the CA, PU probe from the patient.
	Task 64	Discharge the patient.
	Task 65	Turn off the system.

* Tasks marked with an asterisk are those selected as critical tasks.

**Table 4:** The system usability survey questionnaire.

No.	SUS Questions
1	I think that I would like to use this system frequently.
2	I found the system unnecessarily complex.
3	I thought the system was easy to use.
4	I think that I would need the support of a technical person to be able to use this system.
5	I found the various functions in this system were well integrated.
6	I thought there was too much inconsistency in this system.
7	I would imagine that most people would learn to use this system very quickly.
8	I found the system very cumbersome to use.
9	I felt very confident using the system.
10	I needed to learn a lot of things before I could get going with this system.

**Table 5:** The Nasa-TLX questionnaire.

No.	Title	Description
1	Mental demand	How much mental and perceptual activity was required? Was the task easy or demanding, simple or complex?
2	Physical demand	How much physical activity was required? Was the task easy or demanding, slack or strenuous?
3	Temporal demand	How much time pressure did you feel due to the pace at which the tasks or task elements occurred? Was the pace slow or rapid?
4	Overall performance	How successful were you in performing the task? How satisfied were you with your performance?
5	Effort	How hard did you have to work (mentally and physically) to accomplish your level of performance?
6	Frustration level	How irritated, stressed, and annoyed versus content, relaxed, and complacent did you feel during the task?

**Table 6:** Sociodemographic characteristics of participants.

Variable	Option	Frequency
Age	20 s	1
	30 s	8
	40 s	5
	≥60 s	1
Professional Category	Doctor	9
	Nurse	6
Department of participants	Anesthesiology	5
	Emergency	2
	Critical care	8
Clinical Experience *	≥1 and <3 years	1
	≥3 and <5 years	2
	≥5 and <10 years	4
	≥10 years	8
Period of Hemodynamic Device Experience	≥1 and <3 years	2
	≥3 and <5 years	5
	≥5 and <10 years	4
	≥10 years	4
Period of diagnosing using B-lines	No experience	7
	≥1 and <3 years	1
	≥3 and <5 years	4
	≥5 and <10 years	2
	≥10 years	1

* Participants had an average of 10.13 years of clinical experience.

**Table 7:** Task success and failure rates by use scenario.

Use Scenario	Task Success Rate (%)	Task Failure Rate (%)
Check the safety information	95.0	5.0
Prepare for use	91.7	8.3
Register Patient 1	100.0	0.0
CA probe, FA probe attachment	98.9	1.1
Monitoring	92.2	7.8
Alarm setting	84.8	15.2
Fluid responsiveness test	92.2	7.8
Review	84.8	15.2
Discharge Patient 1	100.0	0.0
Prepare pulmonary monitoring	86.7	13.3
Register Patient 2	100.0	0.0
PU probe attachment	97.0	3.0
PU monitoring	93.3	6.7
Discharge Patient 2	97.8	2.2

**Table 8:** Task success and failure rates for tasks 26, 34, 41, 43, and 51 by clinical experience.

Task		Group 1 * (*n* = 7)		Group 2 ** (*n* = 8)	
		Success Rate	Failure Rate	Success Rate	Failure Rate
26	Check the parameter value through the graph of eSVc.	85.71%	14.29%	62.50%	37.50%
34	Pause the alarm.	85.71%	14.29%	62.50%	37.50%
41	To improve hypovolemia, administer 250 mL of colloid and record this intervention.	100.00%	0.00%	50.00%	50.00%
43	Check the fluid responsiveness test record on the review screen.	42.86%	57.14%	75.00%	25.00%
51	Change the parameter’s layout (placement) in order of HRc, eSVc, VTIc, BFc, B-line.	71.43%	28.57%	75.00%	25.00%

* Group 1 consisted of evaluators with less than 10 years of clinical experience. ** Group 2 consisted of evaluators with 10 or more years of clinical experience.

**Table 9:** Statistical analysis results by clinical experience.

Category	Mann–Whitney Test *		Independent Samples *t*-Test **	
	Mann–Whitney U	*p*-Value	t	*p*-Value
Satisfaction	22.5	0.536	-	-
SUS	-	0.147	1.469	0.166
NASA-TLX 1. Mental Demand	18.5	0.281	-	-
NASA-TLX 2. Physical Demand	5	0.006	-	-
NASA-TLX 3. Temporal Demand	17	0.232	-	-
NASA-TLX 4. Performance	25	0.779	-	-
NASA-TLX 5. Effort	10	0.040	-	-
NASA-TLX 6. Frustration Level	-	0.085	−1.961	0.072
NASA-TLX Average	-	0.105	−2.049	0.061

* Mann–Whitney U test for non-normally distributed data. ** Independent samples *t*-test for normally distributed data.

## Data Availability

Data are available from the corresponding author upon request.

## Abbreviations

The following abbreviations are used in this manuscript:

IEC	International Electrotechnical Commission
SUS	System Usability Scale
Nasa-TLX	Nasa-Task Load Index
CA	Carotid Artery
FA	Femoral Artery
PU	Pulmonary Ultrasound
C	Complete
CI	Completed with Issues
NC	Not Complete
